# Association of neighborhood recreational facilities and depressive symptoms among Chinese older adults

**DOI:** 10.1186/s12877-023-04369-0

**Published:** 2023-10-17

**Authors:** Yuexuan Mu, Ming Yi, Qingshuai Liu

**Affiliations:** 1 School of Public health, Soochow University, Jiangsu, China; 2 China University of Political Science and Law, Beijing, China; 3 School of Sociology and Population Studies, Renmin University of China, Beijing, China

**Keywords:** Neighborhood recreational environment, Depressive symptoms, Older adults

## Abstract

**Objectives:**

Neighborhood recreation facilities has been associated with depression that commonly tested with cross-sectional data. This study used longitudinal data to test the effect of neighborhood recreation facilities on the trajectory of depressive symptoms among Chinese older adults.

**Methods:**

Data was derived from the 2014, 2016 and 2018 China Longitudinal Aging Social Survey (CLASS). Depressive symptoms among older adults were obtained using the CES-D scale. The three-level linear growth model of “time point - individual - community” was conducted to test the association between neighborhood recreation facilities and depressive symptoms.

**Results:**

This study consisted of 3,804 respondents living in 333 communities. It was found that community fitness facilities had a significant effect on depressive symptoms in older adults ($\beta$=-1.212, $P<$0.001). A supportive community fitness environment can effectively slow down the rate of increase in depressive symptoms among older adults ($\beta$=-0.415, $P<$0.01). In subgroup analysis, fitness facilities were the important predictor for people in youngest-old group ($\beta$=-1.247, $P<$0.01) and outdoor activity space was a protective predictor for oldest-old people’s depressive symptoms ($\beta$=-0.258, $P<$0.05).

**Conclusions:**

This study demonstrated an association between neighborhood recreation facilities and depressive symptoms in older adults and found the age difference of this effect. Public health department need pay more attention to neighborhood environment construction to promote healthy aging.

## Introduction

China’s population aging is accelerating; 18.7% of its population was 60 and older at the end of 2021. This proportion of the older population has increased by 5.44% compared to ten years ago [1]. In addition, approximately 350 million people worldwide suffer from depression, making it one of the most prevalent mental health disorders. In terms of economic impact, depression ranks among the top five disease categories globally, accounting for 4% of the overall disease burden and 10% of the economic burden associated with non-fatal diseases. The prevalence of depression among older adults in China varies from 11 to 57%, with higher rates observed in individuals over 80 [2]. Thus, preventing and alleviating older people’s mental health disorders has become an urgent health crisis in promoting healthy aging.

As people get older, individuals physical ability decreases while their vulnerability increases, and they become sensitive to the surrounding environment [3]. In addition, life events, such as widowhood and the death of friends, may make them more dependent on the neighborhood environment and community resources [4]. The neighborhood environment is important for shaping a healthy daily lifestyle and social interaction activities among older adults [5].

The neighborhood recreation facilities environment refers to the community places that provide daily activities for older adults to socialize and spend time [6, 7]. According to the Ecological Systems Theory proposed by Bronfenbrenner in 1979 [8], the community environment is considered part of the mesosystem in the model. This involves the interaction between individuals and their living situations [9]. The neighborhood recreation facilities environment is closely connected to an individual’s living environment and is considered a proximal factor that may directly influence an individual’s mental health status [10].

Previous research suggests that older adults engage in social interactions through community recreational facilities [11]. Consequently, they may acquire social resources such as friendship and empathy, establish social networks, and receive social support. These factors can potentially mitigate negative emotions among older adults [12]. Yang found that community-based senior activity centers and open-air fitness equipment significantly predicted depression among older adults [13]. Liu et al. found that the number of recreational facilities reduced depressive symptoms and alleviated loneliness among older adults [14].

Existing studies have validated the correlation between community recreation environments and the mental health of older adults using cross-sectional data. Notably, the Ecological Systems Theory is categorized into microsystems, mesosystems, exosystems, macrosystems, and the dimension of time. However, few studies have focused on how time affects the interaction between people and the environment [15, 16]. Therefore, this study will use longitudinal data to examine the effects of community recreational facility environments on the trajectory of depressive symptom changes in older adults.

Besides, understanding age difference in health effect of neighborhood environment is also a policy entry point for addressing the challenges of growing and aging cities. Specifically, if the negative impact of the community environment on mental health becomes more pronounced as older adults age, then it is important to focus on making age-appropriate environmental changes to improve the mental health of older adults. Neighborhood Sense of Community among residents showed age related difference [17]. Some studies have found that there was significant age difference in the relationship between neighborhood environment and quality of life among older adults [18]. Previous empirical studies have not directly addressed the age difference in the association between the community environment and mental health among older people. Thus, it is important to identify the age difference in neighborhood recreational facilities and trajectory of depressive symptoms.

Neighborhood recreation facilities are less time-consuming and more cost-effective to renovate than major road improvements and pollution treatment projects. The results of this study will assist public health departments in quickly improving older people’s mental health.

## Data and method

### Sample

We obtained the data from the China Longitudinal Aging Social Survey (CLASS), designed by the Institute of Aging, Renmin University of China, and implemented by the China Survey and Data Center, Renmin University of China. This survey covered 29 provinces in China and targeted older adults aged 60 years and older. CLASS conducted a multi-stage probability sampling, dividing the district areas and community levels, with its first nationwide survey completed in 2014 and follow-up surveys every two years. During the baseline survey in 2014, the research project distributed 25 questionnaires in 476 randomly selected communities from 30 primary sampling units (district area), with 11,511 samples obtained in 2014. In 2016, this survey received 11,471 study samples, including 4,553 follow-up samples; in 2018, it obtained 11,418 study samples, including 3,874 follow-up samples. Therefore, the initial sample size for the study can be interpreted as a total of 3,874. This includes 3,874 samples from 2014 (wave 1), 3,874 samples from 2016 (wave 2), and 3,874 samples from 2018 (wave 3).

This study used data from three rounds of follow-up surveys (2014–2018). Three waves of data were pooled together with ID. First, the study excluded samples that responded with “don’t know” or “can’t answer” in the depression symptom survey. Second, the data used in this study are in a three-level nested data format: “time point - individual - community”, and it is necessary to specify the minimum sample size in each level. In a study on " Adequate Sample Sizes for a Three-Level Growth Model,“ researchers found that a sample size of 30 groups, each consisting of 10 individuals, was sufficient for estimating unbiased fixed effects [19]. Most studies support this finding [20, 21]. The sample size of the community in this study was 333 groups, which meets the requirement. Therefore, the study screened the number of individuals at the community level, and communities with fewer than 10 individuals were excluded. In the end, the sample for this study consisted of 1,268 respondents living in 333 communities.

### Measurements

This study’s dependent variable was depressive symptoms. In 1977, Radloff published the CES-D scale containing 20 items [22]. The present study utilizes a subset of the CES-D scale, developed and tested in a study of the living conditions and health status of older adults in Taiwan [23]. The scale consists of nine items in which older adults indicate the frequency of their depressive symptoms in the past week. Each item corresponds to three response options (1 = not, 2 = sometimes, 3 = often). The study reverse-scored the questions on the positive mood to obtain a total score for the nine items on a scale of 9–27. Higher scores indicated more depressive symptoms. The Cronbach′s α coefficient for the scale was 0.82.

Neighborhood recreation facilities was the independent variable. In this study, neighborhood refers to several apartment complex under the jurisdiction of the same neighborhood committee. CLASS asked respondents about the facilities provided in the community to determine whether the community offers a recreational environment. Previous studies concluded that a community recreational environment provides a space for senior fitness and socialization and improves the quality of life for seniors by enhancing their perception of life and promoting emotional relaxation. The premise of these environmental effects is that older adults perceive the community as providing a recreational environment. Therefore, it is more convincing to use older adults’ subjective responses to determine the health effects of the community environment.

Neighborhood recreation facilities was divided into five sections: outdoor activity area, fitness room, library, activity room, chess and card room. Each activity facility was scored on two points (*0 = No; 1 = Yes*).

The control variables included age (youngest-old = 60–74; oldest-old = 75–99), gender (*0 = male; 1 = female*), place of residence (0 = rural; 1 = urban), education level (0 = illiterate; 1 = primary school; 2 = high and middle school; 3 = junior college and above), marital status (*0 = have no spouse 1 = have a spouse*), cohabit with children (*0 = No; 1 = Yes*), income (CNY), self-rated health, and social network (measured by Lubben Social Network Scale 6 (LSNS-6), developed by Lubben et al. [24]).

### Method

Data analyses were performed in Stata 15.0 software. Firstly, descriptive analysis and correlation analysis was performed to describe the main characteristics of older adults. Second, the study performed a three-level linear growth model of “time point - individual - community” to fit the trajectory of depressive symptoms and focuses on examining the role of age and neighborhood recreation facilities on the trajectory of depressive symptoms. In the three-level linear growth model, individual’s observation record is the primary level, the second level of the model include the basic characteristic of older individuals, and the community is the third level.

With the above in mind, we divided three-level linear growth model analysis into four steps: (1) We constructed a null model without any explanatory variables to test whether depressive symptom scores had significant variance across dimensions (model 1). (2) We included the time point and its quadratic term as independent variables in the model to predict the trajectory of depressive symptoms over time (model 2). (3) We included the neighborhood environmental variables and their interaction terms with time point variables in the model to investigate the impact of neighborhood environmental characteristics on individual depressive symptoms (model 3). (4) We performed subgroup analysis to investigate potential differences in the effects of community environment variables on depressive symptoms across different age groups (model 4 and 5).

## Results

### Descriptive analysis

Table 1 illustrated the basic information of all variables. Depressive symptoms increased among older adults from 2014 to 2016 and decreased from 2016 to 2018. The mean score of depressive symptoms was 14.35 in 2018. Each of the neighborhood recreation facilities showed an upward trend over the four-year period. Based on Table 1, the most common recreational facilities in the community are outdoor activity space (56.62%) and activity room (44.72%).

Most people belong to youngest-old group (67.98%). 44.47% of older people are female, and 51.26% live in urban areas. fewer older people were highly educated, with only 8.28% participating in junior college and above. 20.82% of older adults were illiterate. Most older people had a spouse (71.27%), this percentage decreased by 2.1% between 2014 and 2018. More people did not choose to live with their children (69.72%).

**Table 1:** basic information of variables

	2014 ($n$=1268)	2016 ($n$=1268)	2018 ($n$=1268)
Depressive symptoms	13.65$\pm$3.71	15.39$\pm$3.18	14.35$\pm$2.66
Neighborhood recreation facilities			
Activity room	482 (38.13%)	488 (38.49%)	567 (44.72%)
Fitness facilities	194 (15.35%)	107 (8.44%)	262 (20.66%)
Chess and card room	339 (26.82%)	338 (26.66%)	434 (34.23%)
Library	217 (17.17%)	229 (18.06%)	275 (21.69%)
Outdoor activity space	588 (46.52%)	552 (43.53%)	718 (56.62%)
Age			
Youngest-old	1002 (79.02%)	959 (75.63%)	862 (67.98%)
Oldest-old	266 (20.98%)	309 (24.37%)	406 (32.02%)
Gender			
male	704 (55.53%)	704 (55.53%)	704 (55.53%)
female	564 (44.47%)	564 (44.47%)	564 (44.47%)
Education level			
illiterate	264 (20.82%)	264 (20.82%)	264 (20.82%)
Primary school	426 (33.60%)	426 (33.60%)	426 (33.60%)
High and middle school	473 (37.30%)	473 (37.30%)	473 (37.30%)
Junior college and above	105 (8.28%)	105 (8.28%)	105 (8.28%)
Marital status			
Have no spouse	364 (28.73%)	339 (26.74%)	404 (30.83%)
Have a spouse	904 (71.27%)	929 (73.26%)	904 (69.17%)
Place of residence			
Rural	528 (41.67%)	615 (48.50%)	618 (48.74%)
urban	740 (58.33%)	653 (51.50%)	650 (51.26%)
Income (CNY)	9.40$\pm$1.33	10.23$\pm$2.76	9.41$\pm$3.00
Self-rated health			
Very health	153 (12.07%)	117 (9.23%)	78 (6.15%)
Health	423 (33.36%)	520 (41.01%)	467 (36.83%)
Average	396 (31.23%)	414 (32.65%)	530 (41.80%)
Poor	238 (18.77%)	170 (13.41%)	167 (13.17%)
Very poor	58 (4.57%)	47 (3.70%)	26 (2.05%)
Cohabit with children			
No	734 (57.89%)	813 (64.12%)	884 (69.72%)
Yes	534 (42.11%)	455 (35.88%)	384 (30.28%)
Social network	13.62$\pm$6.28	14.44$\pm$5.54	13.57$\pm$5.09

* Categorical variables were described with the number of cases (percentage), and continuous variables were described with mean$\pm$standard deviation

### The effect of neighborhood recreation facilities on depressive symptoms

In this study, a three-level linear growth model of “time point - individual - community” was constructed to fit the trajectory of depressive symptoms among older individuals. In model 1 (Table 2), null model included no independent variables to examine whether there are significant time and community differences in the depressive symptoms. The intra-group correlation coefficient (ICC) of time point was 0.218, indicating that 21.8% of time point variance in depressive symptoms. Besides, there is 4.5% of the community variance in depressive symptom.

Model 2 included the time variable and a quadratic term of the time variable to reflect the nonlinear trend of depressive symptoms. The results revealed a significant increase in depressive symptoms over time. (β_time = 5.900, P < 0.001). Additionally, there was a pattern observed where depressive symptoms initially increased and then decreased as time progressed. (${{\upbeta }}_{{\text{t}\text{i}\text{m}\text{e}}^{2}}$=-1.407, P < 0.001).

Model 3 incorporated community recreational facilities (including Activity room, Fitness facilities, Chess and card room, Library, and Outdoor activity space) and its interaction term with time variables. The results showed that good Fitness facilities had a significant protective effect on initial levels of depressive symptoms in older adults, even after controlling for individual characteristics. Specifically, the presence of better fitness facilities in the community of residence is associated with a decrease in depressive symptoms ($\beta =$-1.212,$P<$0.01). Notably, we also found the interaction term between community fitness facilities and the time variable to be significant ($\beta =$0.415,$P<$0.01). This suggests that although the older population experiences an increase in depressive symptoms over time ($\beta =$6.371, $P<$0.001), good fitness facilities can significantly slow down the rate of increase in depressive symptoms in old age.

Model 4 and Model 5 perform HLM among two samples based on age difference (youngest-old:60–74; oldest-old:75–99). The results showed that fitness facilities were significantly associated with depressive symptoms among youngest old people ($\beta =$-1.247,$P<$0.01). More fitness facilities in the community would decrease the depressive symptoms among youngest old adults. Youngest old people experience an increase in depressive symptoms over time ($\beta =$6.320, $P<$0.001), while good fitness facilities can significantly slow down the rate of increase in depressive symptoms among youngest old people ($\beta =$0.467,$P<$0.01). Among oldest old people, outdoor activity space was significantly associated with depressive symptoms ($\beta =$-0.258,$P<$0.05). More outdoor activity space in the community would decrease the depressive symptoms among oldest old adults. Although we found the significant association between time and depressive symptoms, the interaction term between community fitness facilities and the time variable was not significant.

**Table 2:** The effect of neighborhood recreational facilities on depressive symptoms

	Model 1	Model 2	Model 3	Model 4	Model 5
Intercept	14.512^***^ (0.081)	9.278^***^ (0.495)	8.905^***^ (0.600)	9.744^***^ (0.664)	7.547^***^ (1.048)
Time		5.900^***^ (0.559)	6.371^***^ (0.540)	6.320^***^ (0.599)	6.761^***^ (0.928)
Time^2^		-1.407^***^ (0.138)	-1.511^***^ (0.132)	-1.509^***^ (0.148)	-1.571^***^ (0.219)
Activity room			-0.069 (0.342)	0.200 (0.400)	-0.062 (0.640)
Fitness facilities			-1.212^**^ (0.426)	-1.247^**^ (0.488)	-1.292 (0.870)
Chess and card room			0.163 (0.349)	-0.013 (0.405)	0.686 (0.663)
Library			-0.034 (0.417)	0.009 (0.484)	-0.946 (0.780)
Outdoor activity space			-0.084 (0.296)	-0.172 (0.341)	-0.258^*^ (0.584)
Time*Activity room			-0.100 (0.154)	-0.179 (0.183)	-0.079 (0.275)
Time*Fitness facilities			0.415^**^ (0.191)	0.467^**^ (0.222)	0.537 (0.367)
Time*Chess and card room			0.029 (0.157)	0.098 (0.185)	-0.155 (0.294)
Time*Library			0.028 (0.182)	-0.002 (0.216)	0.287 (0.327)
Time*Outdoor activity space			-0.175 (0.135)	-0.189 (0.160)	-0.080 (0.251)
Var (community)	0.486 (0.188)	0.704 (0.182)	0.427 (0.145)	0.305 (0.158)	0.766 (0.308)
Var (time)	2.330 (0.247)	1.684 (0.202)	1.448 (0.185)	1.567 (0.230)	0.967 (0.373)
Residual	7.866 (0.205)	7.835 (0.202)	6.738 (0.179)	6.744 (0.217)	6.692 (0.423)
AIC	19418.39	19316.63	17985.2	13330.99	4757.64
BIC	19443.37	19354.09	18140.25	13472.55	4874.321

*Coefficient and standard error were reported in five models. Control variables were included in Model 3–5

## Discussion

This study explored the relationship between neighborhood recreation facilities and depressive symptoms among Chinese older adults and identify the age difference in this relationship. Based on the three-level linear growth model of “time point - individual - community”, it was found that fitness facilities significantly associated with depressive symptoms. Fitness facilities was the important predictor for people in youngest-old group and outdoor activity space was a protective predictor for oldest-old people’s depressive symptoms.

This study found that the increase in community outdoor activity space significantly reduced depression symptoms among oldest-old people, which was consistent with previous studies [25, 26]. The open space in the community was the main venue for older adults to perform physical activities and social activities. Neighborhood outdoor activity space was beneficial to improve the daily activities and expand social networks so that it can reduce depressive symptoms [27, 28]. In addition, neighborhood outdoor activity spaces created a sports culture atmosphere for the older adults and improved their psychological status [29].

In this study, the increase of fitness facilities significantly decreased depressive symptoms among youngest-old people. In China, unlike the heavy equipment found in gyms, community public fitness equipment is both convenient and free. This equipment includes twisters, upper body traction machines, spacewalkers, backboards, and low back massagers. Youngest-old people are more capable to participate in exercise than those over 75 years old. Older adults have a narrower social circle and fewer opportunities to exercise, and neighborhood gyms were convenient for them. Previous studies have also found that exercise weekly helps prevent depression in older adults [30].

The findings in this study have some policy implications. Firstly, neighborhood recreation facilities can be included in health assessments of older adults and used to identify older adults with poorer mental health status. Second, the neighborhood age composition is an entry point for policies addressing aging [31]. Combined with our studies, there was age differences in the association between community activity facilities and depression among older adults. Further understanding of the age composition of different communities needs to be tailored to create healthy neighborhood living environments.

Although we obtained some meaningful results, there were some limitations. First, limited by the questionnaire setup of CLASS, objective indicators of neighborhood recreational facilities, such as facility size or quality were not included in this study. Future research needs to measure neighborhood recreational facilities more comprehensively and explore their role in mental health. Second, there is a distinction between presence and use of recreation facilities. This study demonstrated relationship between the presence of recreation facilities and mental health. Future research is needed to compare the effects of frequency of recreational facility use on the mental health of older adults. Third, although we made speculations about the reasons why no neighborhood recreation facilities decreased oldest-old people’s depressive symptoms, future studies can apply qualitative interviews to explore the influence mechanisms further.

## Conclusions

It was found that community with fitness facilities was significantly associated with depressive symptoms. There was a significant age difference in the association between neighborhood recreation facilities and depressive symptoms. Among youngest-old respondents, neighborhood stressors stemmed mainly from fitness facilities, whereas among oldest-aged people, the stressors came from outdoor activity space.

This paper has no Funds. We have no conflicts of interest to disclose.

## Data Availability

On reasonable request, these data may be made available from the corresponding author.
